# Supplementary material for: The Differential Effect of Carbon Dots on Gene Expression and DNA Methylation of Human Embryonic Lung Fibroblasts as a Function of Surface Charge and Dose
Source: Int J Mol Sci. 2020 Jul 4;21(13):4763. doi: 10.3390/ijms21134763 (PMC7369946; doi:10.3390/ijms21134763)

TEM images of nCD (left) and pCD (right)

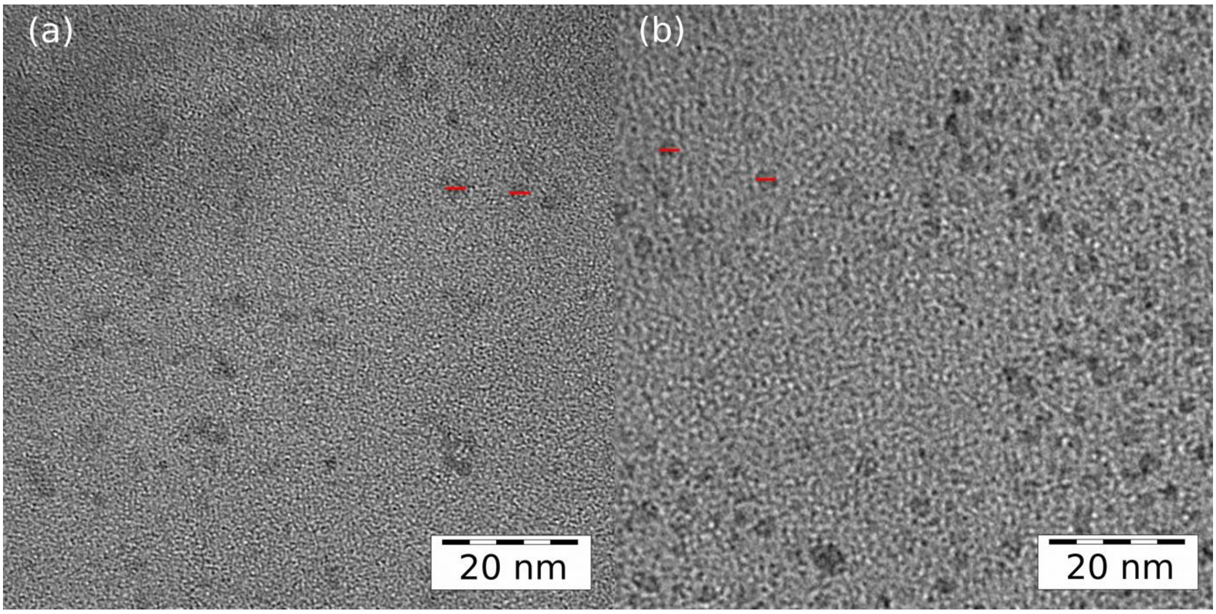

DLS measurement of hydrodynamic diameters for nCD (red) and pCD (green)

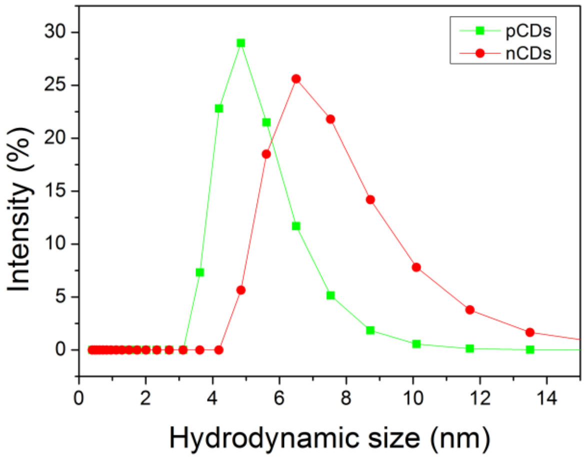

Supplement: Supplementary file 1 [file ijms-21-04763-s001.zip › ijms-833488 rev Supp/Figure S1.pdf]
