# Supplementary material for: The Differential Effect of Carbon Dots on Gene Expression and DNA Methylation of Human Embryonic Lung Fibroblasts as a Function of Surface Charge and Dose
Source: Int J Mol Sci. 2020 Jul 4;21(13):4763. doi: 10.3390/ijms21134763 (PMC7369946; doi:10.3390/ijms21134763)
Supplement: Supplementary file 1 [file ijms-21-04763-s001.zip › ijms-833488 rev Supp/Figure S3.pdf]

Pathways deregulated after pCD and nCD exposure

ID	Source	Pathway name	pCD24h50	nCD4h100	nCD24h10	nCD24h100
1269519	REACTOME	RHO GTPases Activate Formins	X		X	X
1269741	REACTOME	Cell Cycle	X		X	X
138007	Path Int Dat	PLK1 signaling events	X		X	X
1269773	REACTOME	Activation of the pre-replicative complex	X		X	X
1269763	REACTOME	Cell Cycle, Mitotic	X		X	X
1269810	REACTOME	M Phase	X		X	X
1269785	REACTOME	Unwinding of DNA	X		X	X
1269821	REACTOME	Resolution of Sister Chromatid Cohesion	X		X	X
1269768	REACTOME	G1/S Transition	X		X	X
1269772	REACTOME	G1/S-Specific Transcription	X		X	X
137934	Path Int Dat	E2F transcription factor network	X		X	X
1269820	REACTOME	Mitotic Prometaphase	X		X	X
1269784	REACTOME	DNA strand elongation	X		X	X
1269823	REACTOME	Mitotic Metaphase and Anaphase	X		X	X
83054	KEGG	Cell cycle	X		X	X
1269742	REACTOME	Cell Cycle Checkpoints	X		X	X
138080	Path Int Dat	Aurora B signaling	X		X	X
1269777	REACTOME	S Phase	X		X	X
1269779	REACTOME	Synthesis of DNA	X		X	X
1269875	REACTOME	DNA Replication	X		X	X
83039	KEGG	DNA Replication	X		X	X
1269825	REACTOME	Mitotic Anaphase	X		X	X
1269764	REACTOME	Mitotic G1-G1/S phases	X		X	X
1269831	REACTOME	M/G1 Transition	X		X	X
1269509	REACTOME	RHO GTPase Effectors	X		X	X
1269832	REACTOME	DNA Replication Pre-Initiation	X		X	X
1269826	REACTOME	Separation of Sister Chromatids	X		X	X
1269507	REACTOME	Signaling by Rho GTPases	X		X	X
1268690	REACTOME	Eukaryotic Translation Elongation		X		X
1269056	REACTOME	Infectious disease		X		X
1268689	REACTOME	SRP-dependent cotranslational protein targeting to membrane		X		X
1269718	REACTOME	Nonsense Mediated Decay (NMD) independent of the Exon Junction Complex (EJC)		X		X
1268686	REACTOME	GTP hydrolysis and joining of the 60S ribosomal subunit		X		X
1268681	REACTOME	Formation of a pool of free 40S subunits		X		X
1269109	REACTOME	Influenza Life Cycle		X		X
1339156	REACTOME	Selenocysteine synthesis		X		X
1339149	REACTOME	Selenoamino acid metabolism		X		X
1268691	REACTOME	Peptide chain elongation		X		X
1269120	REACTOME	Viral mRNA Translation		X		X
1268688	REACTOME	L13a-mediated translational silencing of Ceruloplasmin expression		X		X
1269115	REACTOME	Influenza Viral RNA Transcription and Replication		X		X
1268679	REACTOME	Eukaryotic Translation Initiation		X		X
1268692	REACTOME	Eukaryotic Translation Termination		X		X
1269108	REACTOME	Influenza Infection		X		X
1268680	REACTOME	Cap-dependent Translation Initiation		X		X
1269765	REACTOME	G0 and Early G1	X		X	
1269799	REACTOME	G2/M Transition	X		X	
1269804	REACTOME	Polo-like kinase mediated events	X		X	
1269797	REACTOME	Mitotic G2-G2/M phases	X		X	
M17370	MSigDB C2	Role of Ran in mitotic spindle regulation	X		X	
1269800	REACTOME	Cyclin A/B1 associated events during G2/M transition	X		X	
M6682	MSigDB C2	CDK Regulation of DNA Replication	X		X	
137925	Path Int Dat	Aurora A signaling	X		X	
1269757	REACTOME	Activation of ATR in response to replication stress	X			X
1269753	REACTOME	G2/M Checkpoints	X			X
1269774	REACTOME	E2F mediated regulation of DNA replication	X		X	
1339113	REACTOME	SUMOylation of DNA replication proteins	X			
1269817	REACTOME	Nuclear Pore Complex (NPC) Disassembly	X			
1269815	REACTOME	Nuclear Envelope Breakdown	X			
1269085	REACTOME	Rev-mediated nuclear export of HIV RNA	X			
1268743	REACTOME	SUMO E3 ligases SUMOylate target proteins	X			
1269095	REACTOME	Interactions of Rev with host cellular proteins	X			
1270037	REACTOME	Cholesterol biosynthesis	X			
SMP00023	SMPDB	Steroid Biosynthesis	X			
142269	BIOCYC	superpathway of cholesterol biosynthesis	X			
PW:0000454	Ontology	cholesterol biosynthetic	X			
1309095	REACTOME	DNA Double-Strand Break Repair	X			
1269688	REACTOME	Processing of Capped Intron-Containing Pre-mRNA		X		
1269654	REACTOME	Transcriptional Regulation by TP53		X		
138045	Path Int Dat	HIF-1-alpha transcription factor network		X		
1269691	REACTOME	mRNA Splicing - Minor Pathway		X		
138057	Path Int Dat	ErbB1 downstream signaling		X		
1268855	REACTOME	Diseases of signal transduction		X		
1269717	REACTOME	Nonsense Mediated Decay (NMD) enhanced by the Exon Junction Complex (EJC)		X		
1270421	REACTOME	Cellular response to heat stress		X		
1269716	REACTOME	Nonsense-Mediated Decay (NMD)		X		
1268682	REACTOME	Formation of the ternary complex, and subsequently, the 43S complex		X		
1383086	REACTOME	Major pathway of rRNA processing in the nucleolus and cytosol		X		
1268684	REACTOME	Translation initiation complex formation		X		
1268685	REACTOME	Ribosomal scanning and start codon recognition		X		
137957	Path Int Dat	LKB1 signaling events		X		
138001	Path Int Dat	mTOR signaling pathway		X		
1383085	REACTOME	rRNA processing		X		
83100	KEGG	Huntington's disease		X		
1269649	REACTOME	Gene Expression		X		
1269443	REACTOME	Signalling by NGF		X		
137940	Path Int Dat	Signaling events mediated by VEGFR1 and VEGFR2		X		
782000	KEGG	Proteoglycans in cancer		X		
83070	KEGG	Adherens junction		X		
83105	KEGG	Pathways in cancer		X		
1270158	REACTOME	Metabolism of amino acids and derivatives		X		
1269655	REACTOME	TP53 Regulates Metabolic Genes		X		
1268683	REACTOME	Activation of the mRNA upon binding of the cap-binding complex and eIFs, and subsequent binding to 43S		X		
1268677	REACTOME	Metabolism of proteins		X		
1268678	REACTOME	Translation		X		
83036	KEGG	Ribosome		X		
946598	KEGG	Thyroid hormone signaling pathway		X		
1268854	REACTOME	Disease		X		
1427846	REACTOME	rRNA processing in the nucleus and cytosol		X		
1270303	REACTOME	Axon guidance		X		
1269376	REACTOME	Basigin interactions			X	
83055	KEGG	p53 signaling pathway			X	
1269709	REACTOME	tRNA Aminoacylation			X	
1269514	REACTOME	RHO GTPases activate CIT			X	
1269838	REACTOME	APC/C-mediated degradation of cell cycle proteins			X	
1383071	REACTOME	TP53 Regulates Transcription of Cell Cycle Genes			X	
1269710	REACTOME	Cytosolic tRNA aminoacylation			X	
MAP00970	GenMAPP	MAP00970 Aminoacyl tRNA biosynthesis			X	
1269837	REACTOME	Regulation of mitotic cell cycle			X	
1269840	REACTOME	Phosphorylation of Emi1			X	
1270244	REACTOME	Extracellular matrix organization			X	
1269796	REACTOME	Removal of licensing factors from origins			X	
137935	Path Int Dat	FOXM1 transcription factor network			X	
1269859	REACTOME	Telomere C-strand (Lagging Strand) Synthesis				X
1269853	REACTOME	Chromosome Maintenance				X
1270350	REACTOME	DNA Repair				X
