# Supplementary material for: The Differential Effect of Carbon Dots on Gene Expression and DNA Methylation of Human Embryonic Lung Fibroblasts as a Function of Surface Charge and Dose
Source: Int J Mol Sci. 2020 Jul 4;21(13):4763. doi: 10.3390/ijms21134763 (PMC7369946; doi:10.3390/ijms21134763)
Supplement: Supplementary file 1 [file ijms-21-04763-s001.zip › ijms-833488 rev Supp/Figure S4.pdf]

Pathways deregulated after pCD and nCD exposure identified based on miRNA-mRNA interactions

ID	Pathway name	pCD24h10	pCD24h50	nCD4h10	nCD4h100	nCD24h10	nCD24h100
hsa00512	Mucin type O-Glycan biosynthesis	X	X	X	X	X	X
hsa04261	Adrenergic signaling in cardiomyocytes	X	X		X	X	X
hsa04921	Oxytocin signaling pathway	X	X		X	X	X
hsa05214	Glioma	X	X		X	X	X
hsa05032	Morphine addiction	X	X		X	X	X
hsa05210	Colorectal cancer	X	X		X	X	X
hsa05205	Proteoglycans in cancer	X	X		X	X	X
hsa05212	Pancreatic cancer	X	X		X	X	X
hsa04724	Glutamatergic synapse	X	X		X	X	X
hsa05213	Endometrial cancer	X	X		X	X	X
hsa04068	FoxO signaling pathway	X	X		X	X	X
hsa04360	Axon guidance		X	X	X	X	X
hsa04024	cAMP signaling pathway	X	X		X		X
hsa04730	Long-term depression	X	X		X		X
hsa05202	Transcriptional misregulation in cancer		X	X	X		X
hsa04810	Regulation of actin cytoskeleton		X		X	X	X
hsa04150	mTOR signaling pathway		X		X	X	X
hsa04919	Thyroid hormone signaling pathway		X		X	X	X
hsa05221	Acute myeloid leukemia		X		X	X	X
hsa04015	Rap1 signaling pathway		X		X	X	X
hsa04310	Wnt signaling pathway		X		X	X	X
hsa04152	AMPK signaling pathway		X		X	X	X
hsa05215	Prostate cancer		X		X	X	X
hsa04151	PI3K-Akt signaling pathway		X		X	X	X
hsa05412	Arrhythmogenic right ventricular cardiomyopathy (ARVC)		X		X	X	X
hsa04510	Focal adhesion		X		X	X	X
hsa04012	ErbB signaling pathway		X		X	X	X
hsa04350	TGF-beta signaling pathway		X		X	X	X
hsa05200	Pathways in cancer		X		X	X	X
hsa04014	Ras signaling pathway		X		X	X	X
hsa05211	Renal cell carcinoma		X		X	X	X
hsa04390	Hippo signaling pathway		X		X	X	X
hsa05220	Chronic myeloid leukemia		X		X	X	X
hsa04550	Signaling pathways regulating pluripotency of stem cells		X		X	X	X
hsa04010	MAPK signaling pathway		X		X	X	X
hsa05218	Melanoma		X		X	X	X
hsa04320	Dorso-ventral axis formation		X		X	X	X
hsa04520	Adherens junction		X		X	X	X
hsa04144	Endocytosis		X		X	X	X
hsa00310	Lysine degradation			X	X	X	X
hsa04512	ECM-receptor interaction			X	X	X	X
hsa05161	Hepatitis B	X				X	X
hsa04066	HIF-1 signaling pathway		X		X	X	
hsa00061	Fatty acid biosynthesis		X		X		X
hsa04710	Circadian rhythm		X		X		X
hsa04070	Phosphatidylinositol signaling system		X		X		X
hsa04141	Protein processing in endoplasmic reticulum		X		X		X
hsa04917	Prolactin signaling pathway		X		X		X
hsa03015	mRNA surveillance pathway		X		X		X
hsa04713	Circadian entrainment		X		X		X
hsa05217	Basal cell carcinoma		X		X		X
hsa04071	Sphingolipid signaling pathway		X			X	X
hsa04915	Estrogen signaling pathway		X			X	X
hsa05020	Prion diseases			X		X	X
hsa05231	Choline metabolism in cancer				X	X	X
hsa00534	Glycosaminoglycan biosynthesis - heparan sulfate / heparin				X	X	X
hsa04722	Neurotrophin signaling pathway				X	X	X
hsa04514	Cell adhesion molecules (CAMs)	X	X				
hsa04723	Retrograde endocannabinoid signaling	X					X
hsa04918	Thyroid hormone synthesis	X					X
hsa04340	Hedgehog signaling pathway		X		X		
hsa04725	Cholinergic synapse		X				X
hsa05100	Bacterial invasion of epithelial cells		X				X
hsa04660	T cell receptor signaling pathway		X				X
hsa04666	Fc gamma R-mediated phagocytosis		X				X
hsa04728	Dopaminergic synapse		X				X
hsa00780	Biotin metabolism			X	X		
hsa04115	p53 signaling pathway				X	X	
hsa00514	Other types of O-glycan biosynthesis				X		X
hsa04540	Gap junction				X		X
hsa00533	Glycosaminoglycan biosynthesis - keratan sulfate				X		X
hsa00604	Glycosphingolipid biosynthesis - ganglio series				X		X
hsa04611	Platelet activation				X		X
hsa05223	Non-small cell lung cancer					X	X
hsa05203	Viral carcinogenesis					X	X
hsa04720	Long-term potentiation					X	X
hsa04530	Tight junction		X				
hsa05131	Shigellosis		X				
hsa01212	Fatty acid metabolism				X		
hsa04920	Adipocytokine signaling pathway				X		
hsa04120	Ubiquitin mediated proteolysis					X	
hsa05222	Small cell lung cancer					X	
hsa04370	VEGF signaling pathway					X	
hsa05166	HTLV-I infection					X	
hsa01040	Biosynthesis of unsaturated fatty acids					X	
hsa05162	Measles						X
hsa04910	Insulin signaling pathway						X
hsa05031	Amphetamine addiction						X
hsa04022	cGMP-PKG signaling pathway						X
hsa00562	Inositol phosphate metabolism						X
hsa04750	Inflammatory mediator regulation of TRP channels						X
hsa05230	Central carbon metabolism in cancer						X
hsa05033	Nicotine addiction						X
hsa00510	N-Glycan biosynthesis						X
hsa04930	Type II diabetes mellitus						X
hsa04210	Apoptosis						X
hsa04668	TNF signaling pathway						X
hsa00250	Alanine, aspartate and glutamate metabolism						X
hsa05160	Hepatitis C						X
hsa04960	Aldosterone-regulated sodium reabsorption						X
hsa04727	GABAergic synapse						X
