# Supplementary material for: The Differential Effect of Carbon Dots on Gene Expression and DNA Methylation of Human Embryonic Lung Fibroblasts as a Function of Surface Charge and Dose
Source: Int J Mol Sci. 2020 Jul 4;21(13):4763. doi: 10.3390/ijms21134763 (PMC7369946; doi:10.3390/ijms21134763)
Supplement: Supplementary file 1 [file ijms-21-04763-s001.zip › ijms-833488 rev Supp/Table S1.docx]

**Table S1.** Elemental composition of pCDs and nCDs according to the XPS analysis with deconvoluted C 1s high resolution spectra.

| Elemental composition (%) | **C 1s** | **O 1s** | **N 1s** | **Cl 2p** |
| --- | --- | --- | --- | --- |
| pCDs | 76.5 | 13.2 | 8.8 | 1.5 |
| nCDs | 63.7 | 20.6 | 15.7 | - |
|  |  |  |  |  |
| High resolution C 1s (%) | **C=C  (284.78 eV)** | **C-O/C-N (286.07 eV)** | **C=O  (287.62 eV)** | **O-C=O  (288.87 eV)** |
| pCDs | 56.2 (284.7 eV) | 33.7 (286.0 eV) | 7.1 (287.6 eV) | 2.9 (288.7 eV) |
| nCDs | 48.1 (284.7 eV) | 24.3 (286.3 eV) | 23.2 (287.6 eV) | 4.4 (289.4 eV) |
